# Designing, construction and characterization of genetically encoded FRET-based nanosensor for real time monitoring of lysine flux in living cells

**DOI:** 10.1186/s12951-016-0204-y

**Published:** 2016-06-22

**Authors:** Seema Ameen, Mohammad Ahmad, Mohd. Mohsin, M. Irfan Qureshi, Mohamed M. Ibrahim, Malik Z. Abdin, Altaf Ahmad

**Affiliations:** Department of Botany, Faculty of Science, Hamdard University, New Delhi, India; Department of Biosciences, Faculty of Natural Sciences, Jamia Millia Islamia, New Delhi, India; Department of Biotechnology, Faculty of Natural Sciences, Jamia Millia Islamia, New Delhi, India; Department of Botany & Microbiology, Science College, King Saud University, P.O. Box 2455, Riyadh, Saudi Arabia; Department of Botany & Microbiology, Faculty of Science, Alexandria University, P.O. Box 21511, Alexandria, Egypt; Department of Biotechnology, Faculty of Science, Hamdard University, New Delhi, India; Department of Botany, Faculty of Life Sciences, Aligarh Muslim University, Aligarh, India

**Keywords:** Fluorescent protein, Fluorescent resonance energy transfer (FRET), Genetically encoded nanosensor, Lysine, Periplasmic binding protein

## Abstract

**Background:**

Engineering microorganisms in order to improve the metabolite flux needs a detailed knowledge of the concentrations and flux rates of metabolites and metabolic intermediates in vivo. Fluorescence resonance energy transfer (FRET) based genetically encoded nanosensors represent a promising tool for measuring the metabolite levels and corresponding rate changes in live cells. Here, we report the development of a series of FRET based genetically encoded nanosensor for real time measurement of lysine at cellular level, as the improvement of microbial strains for the production of l-lysine is of major interest in industrial biotechnology.

**Results:**

The lysine binding periplasmic protein (LAO) from *Salmonella enterica serovar typhimurium* LT2 strain was used as the reporter element for the sensor. The LAO was sandwiched between GFP variants i.e. cyan fluorescent protein (CFP) and yellow fluorescent protein (YFP). Affinity, pH stability, specificity and metal ions effects was scrutinized for the in vitro characterization of this nanosensor, named as FLIPK. The FLIPK is specific to lysine and found to be stable with the pH within the physiological range. The calculated affinity (*K*_*d*_) of FLIPK was 97 µM. For physiological applications, mutants with different binding affinities were also generated and investigated in vitro. The developed nanosensor efficiently monitored the intracellular level of lysine in bacterial as well as yeast cell.

**Conclusion:**

The developed novel lysine fluorescence resonance energy transfer sensors can be used for in vivo monitoring of lysine levels in prokaryotes as well as eukaryotes. The potential of these sensors is that they can be used as reporter tools in the development of metabolically engineered microbial strains or for real-time monitoring of intracellular lysine during fermentation.

**Electronic supplementary material:**

The online version of this article (doi:10.1186/s12951-016-0204-y) contains supplementary material, which is available to authorized users.

## Background

Amino acid industry has occupied an important role in world chemical industries. Annual demand of amino acids used in feed additives and pharmaceutical products is very huge [1]. Every year, more than two million tonnes of l-glutamate [2, 3] and a few thousand tonnes each of l-threonine, l-leucine and l-valine are produced in industries using genetically engineered bacterial strains [4]. The development of strains for the production of l-lysine is of major interest as it is one of the most dominating products with global annual market volume of around 1.5 million tonnes and a predicted market growth of 6–8 % per year [5]. l-Lysine is required as a feed additive for poultry and pig breeding [6, 7]. It improves amino acid balance of the feed component, thereby promoting animal growth and improving meat quality. This action makes possible reduction of protein level of the diet, reducing nitrogen synthesis excretion, and can consistently reduce the cost of feed. l-Lysine along with some other amino acids like aspartic acid is used extensively in pharmaceutical industry in the formulation of diets with balanced compositions; as parenteral protein supplementation during surgical stress; as haematopoietic drug and in amino acid infusion [8, 9]. Thus, lysine is of great industrial importance and is produced mainly from bacterial fermentation. Wild strains of bacteria are able to produce only small amounts of amino acids extracellularly. For large scale production of lysine from microbes, metabolic engineering is being used for developing improved bacterial strain [5]. The metabolic engineering widely encompasses the engineering of the biological systems that process chemicals and materials. Several recent examples highlight the exciting potential of altered biosynthetic pathways in microbial hosts to provide renewable synthesis strategies for commodity chemicals and alternate sources for pharmaceuticals [1, 10–12]. The amalgamation of analytical methods to quantify metabolic fluxes and their control with molecular biological techniques to implement genetic modifications is the basis of metabolic engineering. However, major bottleneck problem in the metabolic engineering is the absence of a suitable tool for real time monitoring of flux of a metabolite in the biosynthetic pathway.

Recent advances in mass spectrometry and nuclear magnetic resonance based measurement of metabolic flux have opened up ways to analyze and screen production strains [13–15]. However, these techniques have a limited temporal and spatial resolution due to disruptive sample preparation and the required sample amount, respectively. Therefore, techniques allowing real time measurement of metabolite changes and variations in local metabolite concentration at the single cell level are beneficial contribution to study flux of metabolites. Genetically encoded FRET-based nanosensors offer the potential to transform information about such a small and specific metabolite into an optical output. These sensors can sense and respond to dynamic levels of metabolites within the host cell, enabling researchers to monitor and optimize natural and introduced metabolic pathways [16, 17]. A range of sensors have been developed for the detection of different metabolites [17–19], however, FRET-based sensor has not been developed so far for the monitoring of lysine at cellular level in living cells.

Here, we developed a series of FRET-based genetically encoded nanosensors which allow the intracellular monitoring of l-lysine at single-cell resolution. These sensors are based on the lysine binding bacterial periplasmic protein (LAO) as a reporter element, which is sandwiched between a donor and acceptor fluorescent protein pair. Cyan fluorescent protein (CFP) and yellow fluorescent protein (YFP) act as donor and acceptor fluorescent proteins, respectively. This study also demonstrated that these nanosensors can be used to measure steady state concentrations and to monitor flux of lysine in bacteria, using microplate fluorescence readers. The beauty of this sensor is that it can be introduced in any cell type and analysis of lysine can be carried out as many times as required in living cell.

## Results and discussion

### Construction of a lysine sensor

**Fig. 1:**
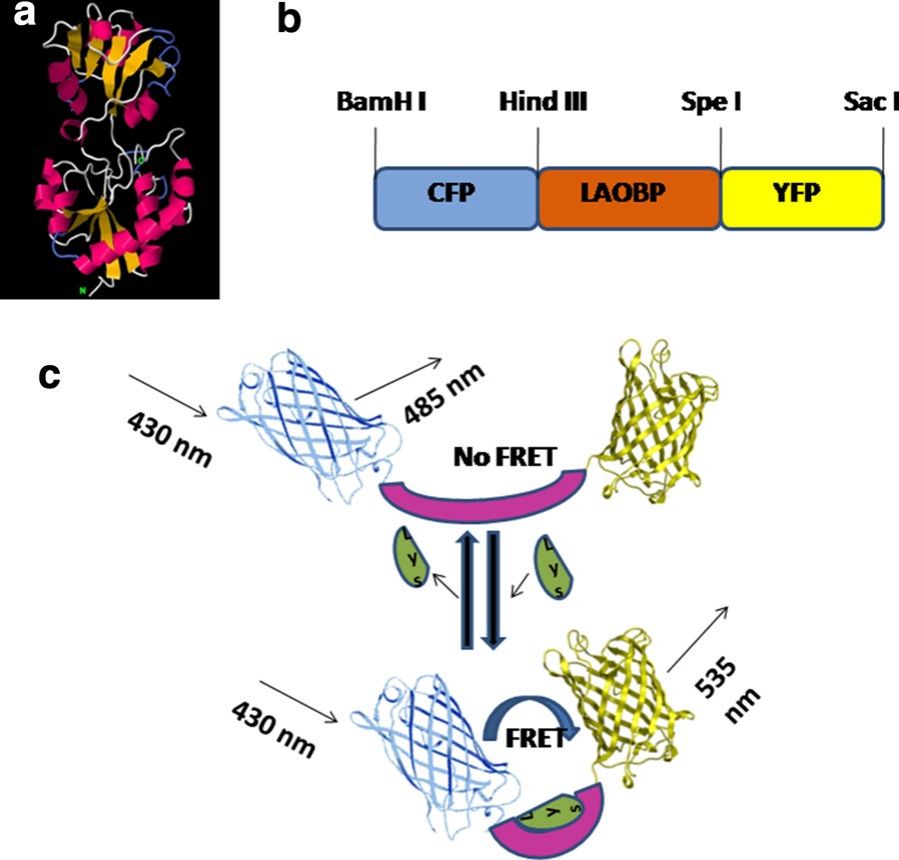
Schematic representation of nanosensor. **a** Ligand free form of LAOBP from *S. typhimurium* showing the N-terminus and C-terminus, **b** linear representation of the nanosensor construct and **c** schematic representation of the lysine induced conformational change in the LAOBP. With the result of binding of lysine to LAOBP, CFP and YFP come closer to each other. Emission of CFP at this stage excites the YFP. Ratio of YFP/CFP emission (FRET ratio) changed as compared to unbound stage

The lysine binding periplasmic protein (LAO) from *Salmonella enterica serovar typhimurium* LT2 strain was used as the reporter element for the sensor. The chosen periplasmic LAO binding protein is a member of the class II (cluster F) binding protein, with both termini on the same protein lobe (Fig. 1a). The LAO is quite different from that of other amino acid-binding proteins in which N and C terminus are present at different lobe. Interestingly, it was found that the LAO closed state reached a higher twisting angle (twist of the C-terminal domain against the N-terminal domain) in the presence of l-lysine than those found for l-arginine and l-histidine [20]. It has been previously shown that such proteins, when fused to FRET donor and acceptor chromophores, can make good sensors as in the case of the *E. coli* glutamate/aspartate-binding protein [16, 21] indicating that other subtle effects (dipole orientation changes, surface interactions between LAO and fluorophores) in addition to changes in distance between the donor and acceptor chromophores contribute to the FRET transfer efficiency. Green fluorescent protein (GFP) variants, CFP and YFP, with different spectral properties were used as fluorescent pair for the construction of nanosensor for real time monitoring of the changes in lysine level. The donor chromophore (CFP) was fused to the N-terminus and an acceptor chromophore (YFP) was attached to the C-terminus of the LAO protein. The constructed nanosensor was named FLIPK. A linear diagram shows the arrangement of the restriction sites in the construct (Fig. 1b). Figure 1c depicts the functional architecture of FRET based sensor.

### In vitro characterization of the nanosensor

**Fig. 2:**
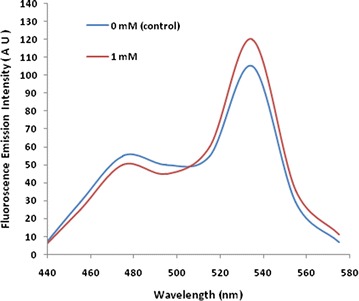
In vitro fluorescence emission spectrum of FLIPK. The fluorescence emission was recorded by excitation at 435 nm in fluorometer without lysine (0 mM) and with lysine (1 mM). The concentration of sensor protein was 0.25 mg/ml

**Fig. 3:**
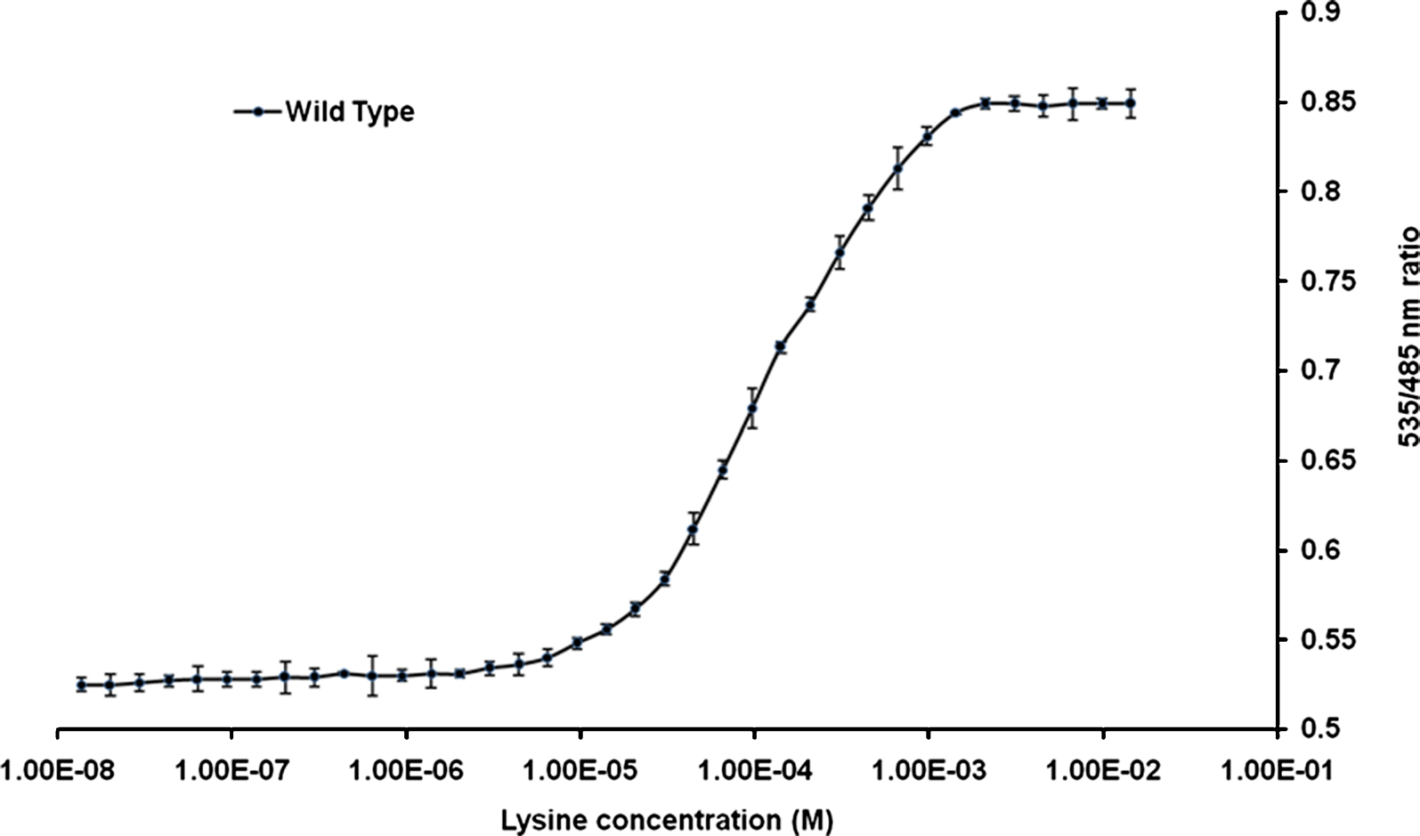
FRET measurement of WT sensor. Lysine titration curve for FLIPK. Purified sensor proteins were diluted with 20 mM PBS buffer. FRET (535/485 nm ratio) was measured at various concentrations of lysine. The concentration of sensor protein was 0.19 mg/ml. Values are means of three independent replicates. *Vertical bars* indicate the standard error

The FLIPK sensor was expressed in *E. coli* BL21 (DE3). The sensor protein was purified by affinity chromatography. Emission spectral analysis showed changes in the respective emission spectra of CFP and YFP after addition of 1 mM lysine showing that the FRET is occurring (Fig. 2). Fluorescence analyses of the purified FLIPK proteins determined the 535 nm/485 nm ratio without lysine as 0.52. By addition of lysine in range of 10^−8^–1 M, the emission ratios for this sensor protein increased, following the sigmoid curves (Fig. 3) and saturated at 1 mM. The calculated affinity (*K*_*d*_) of FLIPK for lysine was 97 µM. Earlier, equilibrium dialysis was used to determine the dissociation constants of different periplasmic binding proteins including lysine. A *K*_*d*_ of 15 nM for lysine was reported by this method [22]. In our experimental work, the affinity calculation was based on FRET ratio change with the conformational change in the chimeric sensor protein. Pre-requisites for the FRET phenomenon are proximity of donor and acceptor chromophores, and overlap between the donor emission spectrum and the acceptor excitation spectrum. Energy is non-radiatively transferred from the donor chromophore to acceptor chromophore. Transfer efficiency is a function of the inverse sixth power of distance between the two chromophores. When the donor and acceptor fluorophores are in close proximity, the emission of excited donor chromophore decreases while emission from the sensitized acceptor chromophore increases. Binding of lysine should brought the N and C termini closer together, thereby increasing FRET. Comparison of liganded and unliganded form of crystal structures of LAO shows that the change in distance between N and C termini translates into a relative movement of the chromophores. The rigid body movement is a rotation of 52° about a virtual axis passing through the two connecting strands, this is a surprisingly large value and is probably the biggest conformational change ever observed [23]. This movement affect the relative orientation of the transition dipoles of attached chromophores and lead to a change in FRET. As the developed FRET based sensor shows maximum FRET ratio change in terms of conformational change on lysine binding as compared to arginine, therefore different affinity was expected for lysine.

**Fig. 4:**
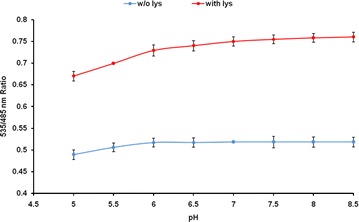
pH stability of the FRET signal of FLIPK. YFP/CFP emission ratios were measured in PBS buffer with different pH, in the absence (*blue*) and in the presence (*red*) of 1 mM of lysine. Stability increases with pH while the sensor stabilizes above pH 7.0. Concentration of sensor protein was 0.16 mg/ml. Values are means of three independent replicates. *Vertical bars* indicate the standard error

**Fig. 5:**
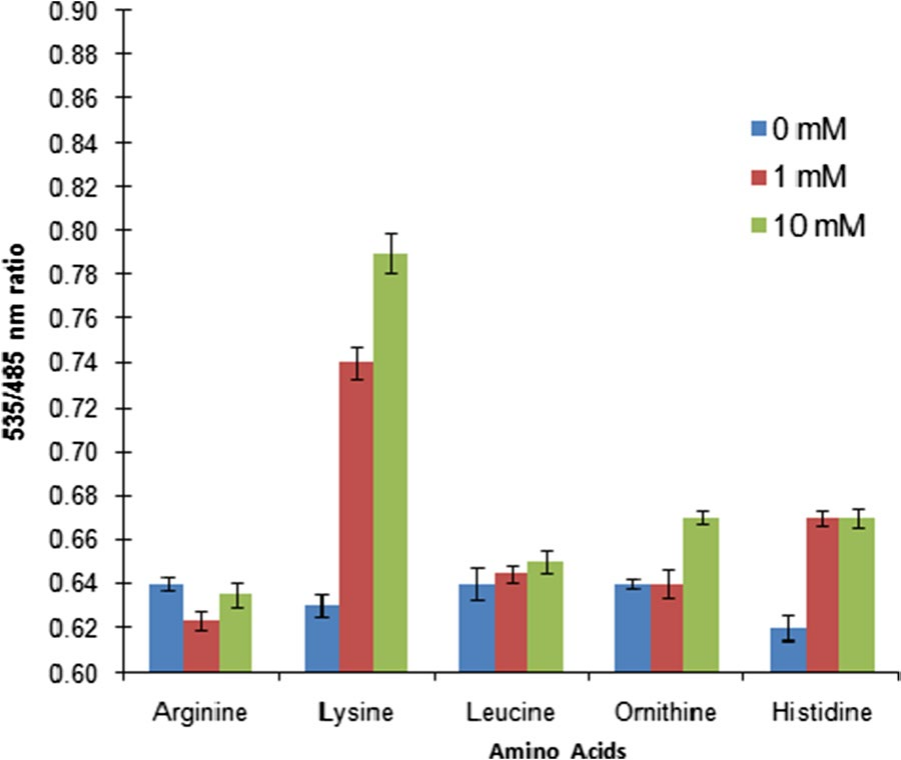
In vitro analysis of FLIPK (WT) nanosensor. Ligand specificity of the FLIPK. Concentration of sensor protein was 0.20 mg/ml. Values are means of three independent replicates. *Vertical bars* indicate the standard error

To check the buffer and pH stability of the FRET signal of FILPK, the fluorescence emission intensities were tested in various buffer system and pH range of 5.0–8.5 in the presence and absence of lysine. The FLIPK sensor showed maximum efficiency in PBS buffer among the different buffer systems used (Additional file 1: Figure S1). With the different pH of PBS buffer, sensor protein showed sensitivity towards acidic pH with FRET ratio increasing up to pH 7.0 but in alkaline pH stability of the FRET signal of the sensor protein increased with slight change in the FRET ratio (Fig. 4). These experiments demonstrated that there is no remarkable effect of pH change on the affinity of FLIPK and pH 7.5 was considered suitable for FLIPK. Therefore, PBS buffer at pH 7.5 was selected for further experimental analysis as the FRET signal of the sensor found to be stable at pH 7.5 which is in the range of physiological pH. The pH stability of the FRET signal of these nanosensors makes them more convenient to monitor the level of lysine in vivo. The strength for engineering the FRET-based nanosensor for metabolites depends upon the sensor domains that undergo conformational changes that are large enough to translate metabolite binding into a change in FRET [24]. The FLIPK meets this criterion as there was a sufficient change in the YFP/CFP emission intensities with addition of lysine. Intensity-based approach for the measurement of FRET ratio between donor and acceptor intensity has been utilized earlier in developing the sensors [17, 25–27]. To check specificity towards lysine, the FLIPK sensor protein was tested with leucine, arginine, ornithine and histidine at concentrations of 1 and 10 mM each. FLIPK showed maximum specificity towards lysine with significant increase in the FRET ratio. The non-significant change in FRET ratio was observed with other amino acids (Fig. 5).

### Affinity mutants of the sensor

**Fig. 6:**
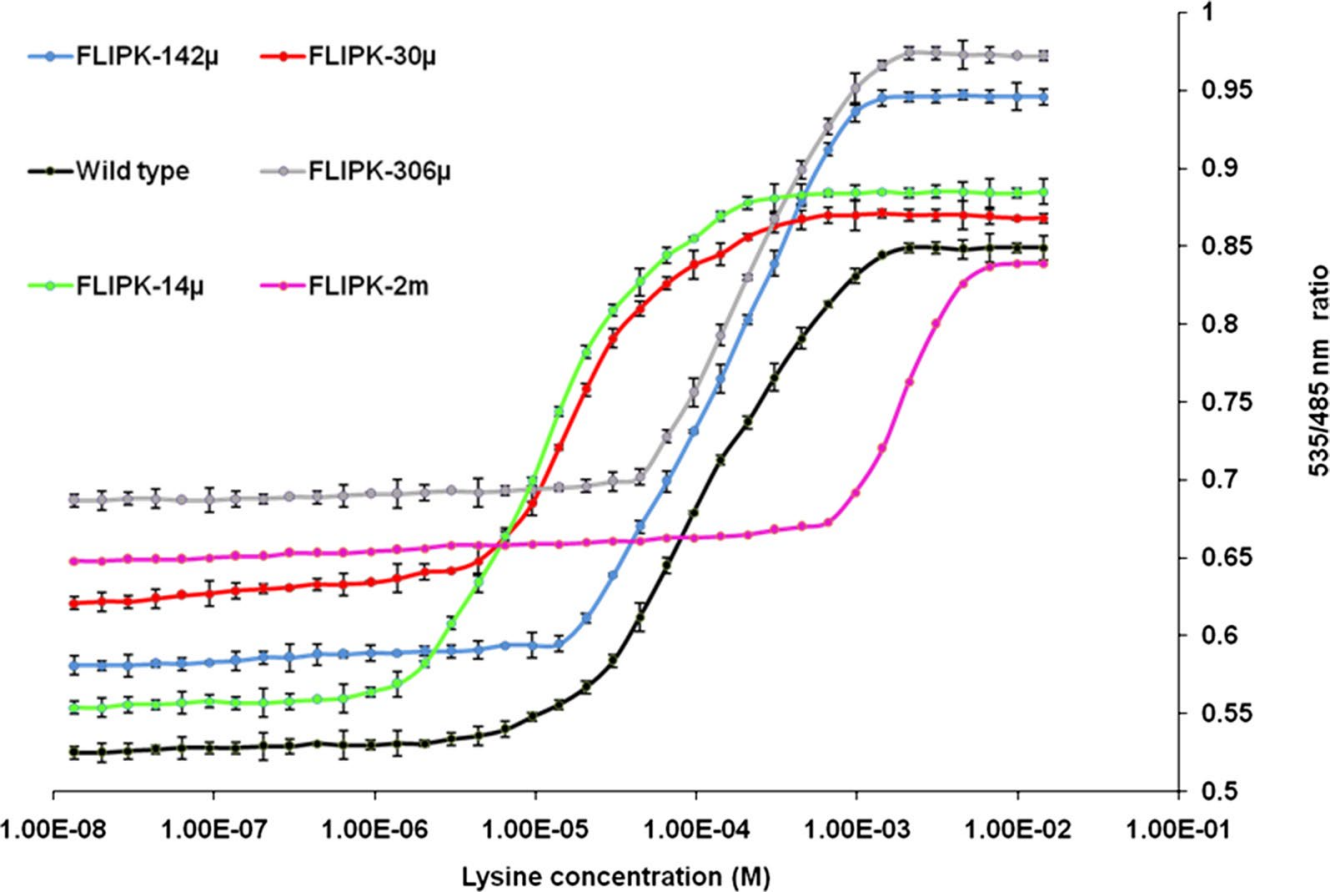
In vitro ligand dependent FRET ratio change of FLIPK in the presence of l-lysine. Affinity mutants Y14A, R77L, F52A, D161I and S72A were developed. Mutations at position 14 tyrosine substituted by alanine (Y14A), arginine 77 to leucine (R77L), phenylalanine 52 to alanine (F52A), and aspartic acid 161 to isoleucine (D161I) and serine at 72 position by alanine (S72A). Concentration of sensor protein was 0.24 mg/ml. Values are means of three independent replications. *Vertical bars* indicate standard error

To expand the physiological range for detection of lysine, a set of sensors were generated by site directed mutagenesis of the amino acid residues involved in lysine binding. LAO bind lysine via hydrogen bonding and the two aromatic rings of Tyr-14 and Phe-52 sandwich lysine by utilizing charged side chains (Arg-77, Asp-161, Asp-11) and hydroxyl group Ser-72 to stabilize the alpha-ammonium and alpha-carboxylate groups [28]. By introducing point mutations, a total of five affinity mutants were developed (Additional file 2: Figure S2) with differing binding constants, providing a set of nanosensors with a broad concentration range between 2 µM to 4.5 mM. The calculated *Kd* of WT, S72A, Y14A, R77L, D161I, F52A were 97, 30, 142, 306, 2000 and 14 µM respectively (Table 1, Fig. 6). Maximal ratio change of 0.97 was observed by the purified sensor FLIPK-306µ (R77L) upon addition of lysine. The normal range of plasma lysine level in an adult is 61–119 μM [29]. Limit of detection range of the FLIPK and mutants (i.e. 2 µM to 4.5 mM) proves the concept of measuring the physiological level of lysine. Developing the mutants enhanced detection range of the sensors and are useful to measure lysine level at different physiological scales.

**Table 1:** FLIPK affinity mutants

Sensor name^a^	Sequences	*K*_*d*_ (µM)	Dynamic range^b^ (µM)
FLIPK-97µ	Wild type	97	14–980
FLIPK-30µ	S72A	30	4.0–208
FLIPK-142µ	Y14A	142	20–1000
FLIPK-306µ	R77L	306	65–1500
FLIPK-2 m	D161I	2000	1000–4500
FLIPK-14 µ	F52A	14	2.0–150

Binding constants were determined in vitro

^a^Number next to the mutant name stands for the Kd determined for the sensor variants

^b^ Effective quantification range between 10 and 90 % saturation of the sensor

### In vivo characterization of the nanosensor in bacteria

**Fig. 7:**
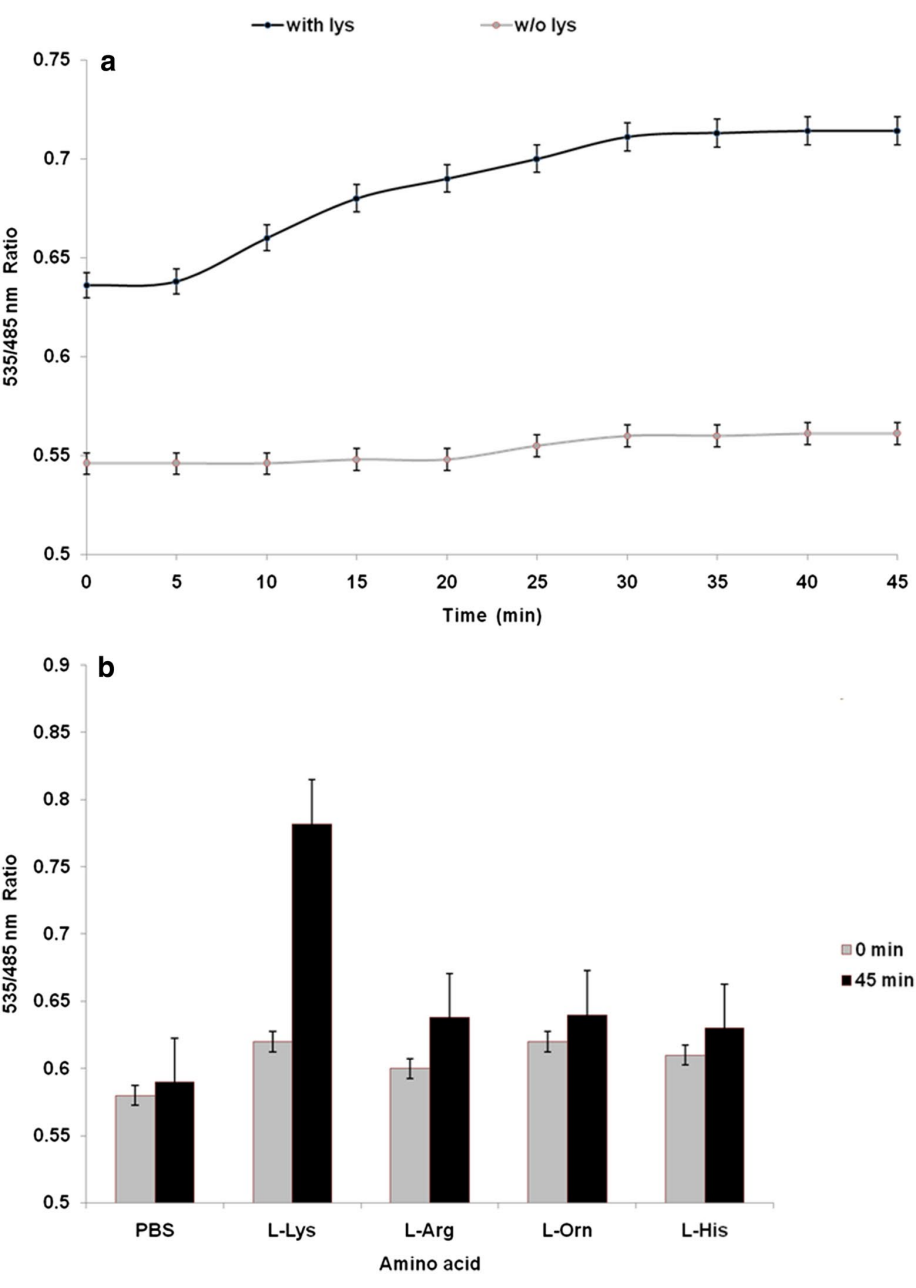
In vivo analysis of the FLIPK (WT) nanosensor. **a** FRET ratio change in response to the 10 mM lysine (*black circles*) and without lysine (*grey circles*) in 20 mM PBS buffer. **b** FRET (535/485 nm) ratio of the bacterial cell suspension before and after incubation with various amino acids for 45 min

In order to examine the function of the FLIPK sensor in live cells, the sensor was expressed in the cytosol of bacterial cells. The suspension of the *E. coli* BL21 (DE3) bacterial cells were tested with and without lysine. Addition of lysine externally showed changes in the FRET ratio significantly. No significant change in FRET ratio was observed in the absence of lysine. The FRET ratio of the cell suspensions increased distinctly with lysine after 5 min and saturated at 45 min (Fig. 7a). As the FRET based sensors quantify changes in intracellular concentrations of ligand in living cell, it has significant applications for fermentation processes in the food, pharmaceutical and cosmetic industries. In this study, lysine FRET nanosensor was used to quantify intracellular lysine concentrations in bacterial cell cultures in a 96-well microplate fluorimeter. These results demonstrate that FLIPK responded the changes in concentration of lysine with time when external lysine was added to FLIPK-transformed bacterial cells. This is constant with the uptake of these amino acids into cytosol of *E. coli*. A group of low and high affinity system catalyzes the competent uptake of amino acids into cytoplasm of bacteria [30]. The uptake of lysine was observed to be saturated within 40–45 min at 10 mM that demonstrate the accumulation of the lysine inside the bacterial cell. On the other side impact of other amino acids was tested by adding them manually. PBS buffer, arginine, ornithine and histidine did not change the FRET ratio but a major change was observed with lysine (Fig. 7b). These results showed in vivo specificity of the sensor towards lysine.

### In vivo imaging of uptake of lysine

**Fig. 8:**
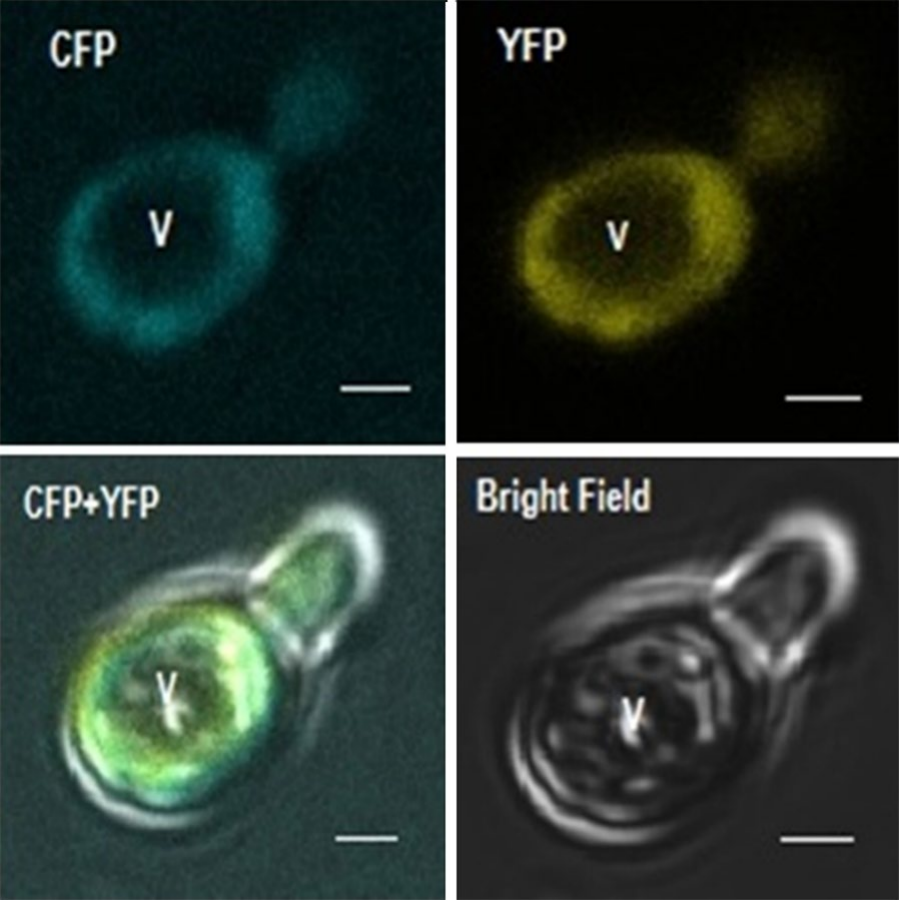
Confocal imaging of FLIPK (WT) expressing yeast cell. FLIPK is detected in the cytosol, whereas no signal was found in the vacuole (V). (*Bar* = 1 µm)

**Fig. 9:**
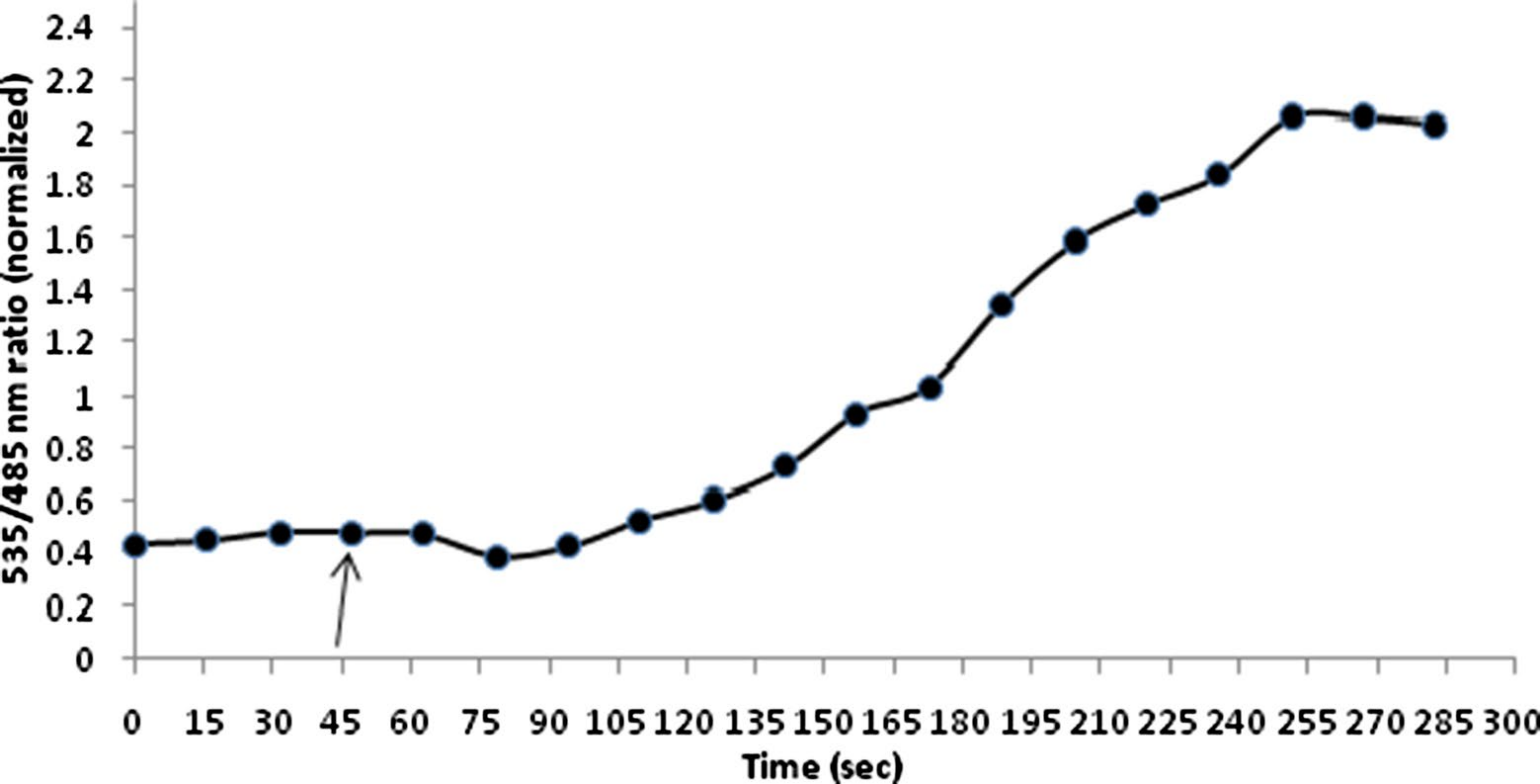
Lysine concentration change in the cytosol of yeast. *S. cerevisiae*/URA3 strain BY4742 expressing the sensor FLIPK. The *graph* indicates the emission intensity ratio (535/485 nm ratio) for a single yeast cell. Addition of 100 mM lysine (shown by *arrow*) increased the ratio by 2.0

The activity of FLIPK nanosensors was also tracked in live bacterial and yeast cells. Bacterial cells were grown to express the chimeric sensor protein and images were taken (Additional file 3: Figure S3). Confocal images of the bacterial cells showed that the bacteria were successfully expressing FLIPK sensor protein. Environmental sensitivity of the fluorescent proteins can generate disruption in specificity of nanosensor [31]. With parallel use of same fluorophores with various amino acids ligand binding proteins can eliminate the effect of environment. The developed lysine sensors showed high responsive FRET changes in in vivo study of bacteria. Yeast was chosen as eukaryotic model system to study the uptake mechanism of lysine and confocal images of expressed yeast cells were also taken. Imaging was carried out by using Leica confocal microscope. Confocal imaging of FLIPK expressing yeast cells showed that the fluorescent chimeric protein is expressed in cytosol, whereas no signal was detected in vacuole (Fig. 8) proving the concept of expression of FLIPK in any cell type. Thus, FLIPK should allow direct monitoring of lysine uptake into cytosol with a subcellular resolution. On addition of 100 mM extracellular lysine, the 535 nm/485 nm emission intensity ratio increased by 2.0, indicating that lysine is transported into yeast cytosol, where it is recognized by FLIPK (Fig. 9 and Additional file 4: Figure S4 and Additional file 5: Figure S5). The developed nanosensor responded to changing level of lysine specifically. Imaging data showed that the 535 nm/485 nm ratio increased sharply up to 255 s after addition of lysine and then it reached to saturation. Live cell imaging of the yeast cells shows that the FLIPK sensor is effectively working in the eukaryotic cells. Developed nanosensor responded to the dynamic level of lysine specifically.

## Conclusions

In the present study, the LAO binding protein was converted successfully into a genetically encoded FRET based lysine nanosensor. Mutagenesis allowed the generation of affinity mutants covering a broad range of physiologically relevant lysine concentrations. The developed sensors were used to visualise and measure lysine level both in vitro and in vivo. The data shown here demonstrate that the FRET based nanosensor can be used in prokaryotes as well as in eukaryotes. This study also paves the road to elucidate compartment-specific dynamics and regulatory events in higher cells that involves the lysine metabolism. The nanosensors will be useful for a wide range of applications, such as studying lysine uptake and identifying the efflux mechanism. Obviously, a set of novel nanosensors will offer us with tools for scientific, medical, and environmental applications. Furthermore, the transgenic organisms expressing FLIPK can be used for measuring the lysine level in plants and in animals, as demonstrated for the arginine FRET indicator in *Arabidopsis thaliana* roots [32] and glutamate in animal cells [16]. The potential of these sensors can be used as reporter tools in development of metabolically engineered microbial strains for the real time monitoring of metabolites during fermentation process.

## Methods

### Fluorescent indicator protein (FLIP) constructs and plasmids

The reporter element LAO, a periplasmic binding protein, was found from *Salmonella enterica serovar typhimurium* LT2 strain. The protein was flanked by CFP and YFP. CFP and YFP sequences were taken from pDH18 vector (generously provided by Yeast Resource Center, Washington, USA) and amplified by polymerase chain reaction after shortening the 5′ ends and 3′ ends to four codon and two codon each, respectively. The CFP and YFP sequences were cloned into the pGEM-T easy vector (Promega, USA). LAO sequence was retrieved from a National Center for Biotechnology Information (NCBI) database and structure of LAO protein was retrieved from the Protein data Bank (PDB). The gene (argT) encoding LAO was amplified by PCR from genomic DNA of *Salmonella enterica serovar typhimurium* LT2 strain. The first 22 amino acids (signal peptides) were removed by the help of a SignalP 4.1 server (CBS, Denmark). All oligonucleotides used were synthesized from Sigma (USA). The forward primer P1 used was 5′-CCCAAGCTTGGGGCGCTCCCGCAAACGGTTCGT-3′ containing a restriction site *Hind*III and the reverse primer P2 was 5′-CGGACTAGTCCGATCGCCGTAAACATT-3′ with restriction site *Spe*I. The underlined sequences show the restriction sites. STOP codon was removed from the 3′ end. Amplified LAO fragment was then inserted into the CFP-YFP cassette with appropriate restriction sites, yielding the CFP-LAO-YFP construct in pGEM-T easy vector. The chimeric sequence was excised at *Bam*HI and *Sac*I sites from pGEM-T easy vector and sub-cloned into the bacterial expression vector, pRSET-B (Invitrogen, USA). This adds an in frame (His)_6_ tag at the amino terminus of the protein sequence. The construct was named as FLIPK (fluorescent indicator protein for lysine). This construct (pRSET-CFP-LAO-YFP) was introduced into the *E. coli* BL21 (DE3) by electroporation. To confirm fidelity, the chimeric fragment was sequenced (Additional file 6: Figure S6).

Previously, fluorescent dyes have been used in the development of such sensors [19]. The reason for replacing dyes with fluorescent proteins was their limitations in the living cells like difficulty in introducing such dyes into living cells and their toxicity to cells [21]. To facilitate the effortless construction of this nanosensor, a phage λ recombination vector based on pRSET-B was used for terminal fusion of target genes to the fluorescent protein variants under control of T7 promoter. The fusion protein carries the N-terminal (His)_6_-affinity tag for purification from *E. coli* cell-free extracts. The pRSET-B vector contains the ampicillin resistance β-lactamase gene and the phage f1origin of replication for site-directed mutagenesis. For the expression of the sensor protein in yeast, FLIPK sequences were transferred to the destination vector (pYES-DEST52) via pENTR4 vector using gateway cloning strategy that resulted into vector pYEST-DEST-FLIPK sequentially, which encodes attR1 site-chloramphenicol acetyl transferase gene- ccdB gene-attR2 gene.

### Expression and purification of FLIPs

The pRSET-CFP-LAO-YFP transformed *E. coli* BL21 (DE3) were grown at 20 °C for 24 h up to OD_600_ 0.6. The expression of the nanosensor gene construct was induced by adding 1 mM isopropyl β-D-1-thiogalactopyranoside (Fermentas, Germany) and transformed bacterial cells were grown at 20 °C for 48 h in the dark for the production of nanosensor protein. Then the bacterial cells were harvested by centrifugation at 6500×*g* for 20 min at 4 °C in refrigerated centrifuge (HERMLE Labortechnik GmbH, Germany) and resuspended in 20 mM Tris–Cl (pH 8.0). The cells were disrupted by ultrasonication (Sonics, USA). Purification of sensor protein was done using the Ni–NTA His-tag affinity chromatography resin (Novagen, USA). The binding of the sensor protein to the resin was done for 4 h at 4 °C, washed in a column with 20 mM Tris–Cl and 10 mM imidazole (pH 8.0) and then eluted with 20 mM Tris–Cl and 50 mM imidazole. The protein was quantified by Bradford’s method using bovine serum albumin as standard [33]. The purity and molecular weight of the sensor proteins was confirmed by sodium dodecyl sulfate polyacrylamide gel electrophoresis (SDS-PAGE, with 12 % acrylamide) analysis by the method of Laemmli [34] (Additional file 7: Figure S7).

### In vitro characterization of the sensor protein

Characterization of sensor protein was initially carried out in different buffer systems and pH value. For this study 20 mM of each PBS [NaCl (100 mM), KCl (2.7 mM), Na_2_HPO_4_ (16.2 mM), KH_2_PO_4_ (3.8 mM)], Tris–HCl, TBS [Tris–Cl (50 mM), NaCl (100 mM)], 3-(N-morpholino) propanesulfonic acid (MOPS), phosphate buffer and HEPES buffer was taken. The eluted sensor protein was diluted 20 times by the respective buffers. It was found that the sensor protein showed least variation in FRET ratio (535 nm/485 nm) with different pH of PBS buffer. Stability of the FRET signal was also measured with PBS buffer pH range 5.0–8.5 in presence of lysine (1 mM) and in the absence of lysine. The change in the fluorescence resonance energy transfer (FRET) ratio with respect to change in buffer and pH was monitored using a monochromator microplate reader (DTX880, Beckman Coulter, USA). The emission spectra were recorded by using a fluorometer (LS50B Perkin Elmer, USA) in 20 mM PBS buffer (pH 7.5). Fluorescence emission spectra were taken by exciting the CFP at 430 nm and then the emission was observed in the range of 450–580 nm.

Specificity of the sensor protein was measured by using five amino acids, arginine, leucine, lysine, ornithine and histidine at three different concentrations i.e. 0, 1.0 and 10 mM, each. FRET measurement was carried out by using a microplate reader. The excitation filter/slit used was 430 nm/20 nm and emission filters/slits for CFP and YFP were 485 nm/20 nm and 535 nm/25 nm, respectively. 180 µl of diluted sensor protein and 20 µl of substrate were taken in different well. Affinity of sensor protein was monitored by using different concentrations of lysine. All analysis was performed in 20 mM PBS buffer (pH 7.5).

To determine the *K*_*d*_ of the FLIPK sensors, sensor protein was mixed with different concentrations of lysine in 20 mM PBS buffer (pH 7.5) and YFP/CFP ratio was recorded. By using the ratio change on ligand binding, binding constants (*K*_d_) were determined by fitting ligand titration curve in a simple binding isotherm: ${\text{S}} = (r - r_{\text{apo}} )/(r_{\text{sat}} - r_{\text{apo}} ) = \left[ L \right]/\left( {K_{d} + \left[ L \right]} \right)$, where S is saturation; [*L*] is ligand concentration; *r* is ratio; *r*_apo_ is ratio in the absence of ligand and *r*_sat_ is ratio at saturation with ligand. All analyses were carried out with three independent protein preparations. FRET was used to characterize the sensor protein with various parameters. The FRET ratio was determined as fluorescence intensity at 535 nm (YFP) divided by fluorescence intensity at 485 nm (CFP).

### In vivo measurement of lysine

In vivo analysis was performed by following the earlier protocols [35, 36]*. E. coli* BL21(DE3) cells were transformed with pRSET-FLIPK, which encodes a lysine FLIP nanosensor with a *K*_*d*_ of 97 µM. Cultures were incubated in Luria Bertoni broth in baffled Erlenmeyer flasks for 48 h in the dark at 20 °C temperature in shaker incubator and then stored at 4 °C for overnight to ensure the proper folding of the fluorescent proteins. Then starved of nitrogen in M9 medium at 37 °C for 2 h and adjusted to OD_600_ 3.5 and kept in 20 mM PBS buffer solution at pH 7.5. Then 180 µl of cells were dispended in the wells of microplate. 20 μl of 10 mM lysine in nitrogen starved medium grown cells were added and the fluorophores emissions were recorded for 45 min. For monitoring the accumulation rates, the injection module of microplate reader was used. Similar procedure was followed with other amino acids (arginine, ornithine, histidine) to see the specificity of FLIPK.

### Monitoring of lysine in yeast cell

For expression of FLIPK, *S. cerevisiae*/URA3 strain BY4742 was used as the eukaryotic host system. The yeast cells were grown and transformed by pYES-DEST-CFP-LAO-YFP vector in which the expression of CFP-LAO-YFP gene was controlled by GAL1 promoter. The transformed yeast cells were grown for 3–5 days in Synthetic Defined (SD)-growth and 1 % galactose as inducer. Imaging of expressed yeast cells were performed on confocal microscope (Leica DMRE) equipped with a confocal head TCS-SPE (Leica, Wetzlar, Germany). The yeast cells were fixed on glass slides by using medical adhesive rinsed with the growth medium and covered by a cover slip. To measure the lysine in yeast cells, dual emission intensity ratio was recorded by using LAS-AF software (Leica, Wetzlar, Germany) with 436 nm/20 nm excitation, two emission filters i.e. 480 nm/40 nm for CFP and 535/30 nm for YFP. For recording the data, FRET sensitized emission tool without background subtraction of the Leica confocal software. The data was further processed by using Adobe Photoshop. Images of single cell and multiple cells were captured by selecting the region of interest (ROI). Similar procedure was followed for the imaging of expressed bacterial cells.

### Generation of affinity mutants

To increase the physiological range of lysine sensor, affinity mutants were developed. Point mutations were generated to change the affinity of sensor by using quick change site directed mutagenesis kit (Stratagene, USA). Various affinity mutants were developed by substituting the amino acid residues in binding pocket of LAO binding protein. Mutations at position 14 tyrosine substituted by alanine (Y14A), arginine 77 to leucine (R77L), phenylalanine 52 to alanine (F52A), and aspartic acid 161 to isoleucine (D161I) and serine at 72 position by alanine (S72A). All mutants were expressed and purified as the wild type for further analysis.

## Additional files

10.1186/s12951-016-0204-y pH stability of the FRET signal of FLIPK. Fluorescent intensity ratio of sensor protein is more stable with PBS buffer whereas in other buffers it is fluctuating. Sensor protein was prepared in Tris-HCl buffer, Phosphate buffer (PB), Tris-Buffered Saline (TBS), Phosphate-BufferedSaline (PBS), HEPES buffer and MOPS buffer at pH 5-8.5 as indicated in the absence of lysine (A) and in the presence of lysine (1 mM) (B). Values are means of three independent replicates. Vertical bars indicate the standard error.
10.1186/s12951-016-0204-y Redesign of lysine-binding site for a wide physiological range of detection of lysine and an enhanced response. Predicted structures of different binding site variants in LAOBP. Mutated residues are shown in yellow: Y14A, R77L, F52A, S72A and D161I.
10.1186/s12951-016-0204-y Confocal imaging of bacterial cells [*E. coli* BL21 (DE3)] expressing the FLIPK.Scale bar 5 μm. Sensor protein production in the bacterial cells and CFP, YFP and CFP+YFP merged indicating the specific excitation and emission wavelength of the fluorophores. Dual emission intensity ratio was recorded by using LAS-AF software (Leica, Wetzlar, Germany) with 436 nm /20 nm excitation, two emission filters i.e. 480 nm /40 nm for CFP and 535/30 nm for YFP.
10.1186/s12951-016-0204-y Confocal imaging of yeast (*S. cerevisiae* /URA3 strain BY4742) with the time. After addition of 100 mM lysine (indicated by an arrow) the intensity of YFP increases and there is decrease in CFP emission intensity showing that the lysine is transported into the yeast cytosol, where it is recognized by FLIPK.
10.1186/s12951-016-0204-y Non-normalized and normalized data of visual dynamic of lysine concentration change in the cytosol of yeast.
10.1186/s12951-016-0204-y A. Nucleotide sequences of construct with His-tag (green)- CFP (cyan) –LAO (grey) –YFP (yellow). B. Amino acid sequence of FLIPK construct.
10.1186/s12951-016-0204-y SDS-PAGE of expressed FLIPK in *E. coli* BL21 (DE3). M= protein marker; L1= uninduced protein sample; L2= induced protein sample.

## Abbreviations

GFP: green fluorescent protein
CFP: cyan fluorescent protein
YFP: yellow fluorescent protein
FLIP: fluorescent indicator protein
PBP: periplasmic binding protein

## References

[1] Ma SM, Li JWH, Choi JW, Zhou H (2009). Complete reconstitution of a highly reducing iterative polyketide synthase. Science.

[2] Kimura E, Eggeling L, Bott M (2005). l-Glutamate production. Handbook of *Cornybacterium glutamicum*.

[3] Hanbler E, Burkovski A, Burkovski A (2008). Molecular mechanisms of nitrogen control in corynebacteria. *Corynebacteria*: genomics and molecular biology.

[4] Eggeling L, Ratledge C, Kristiansen B (2001). Amino acids. Basic biotechnology.

[5] Becker J, Wittmann C (2012). Systems and synthetic metabolic engineering for amino acid production—the heartbeat of industrial strain development. Curr Opin Biotechnol.

[6] Hirose Y, Shibai H, l-Glutamic acid fermentation, In: Comprehensive biotechnology 3, Moo-Young M., editor 1985, p. 595–600.

[7] Eggeling L, Sahm H (1999). l-glutamate and l-lysine: traditional products with impetuous developments. Appl Microbiol Biotechnol.

[8] Hirose Y, Okada H, Microbial production of amino acids, In: Microbial technology 1, Peppler HJ, Perlman D, editors 1979, p. 211–40.

[9] Costa Ferreira M, Duarte JC (1992). Amino acid accumulation by an analogue—sensitive mutant of *Corynebacterium glutamicum*. Biotechnol Lett..

[10] Atsumi S, Hanai T, Liao JC (2008). Non-fermentative pathways for synthesis of branched-chain higher alcohols as biofuels. Nature.

[11] Ro DK, Paradise EM, Ouellet M, Fisher KJ (2006). Production of the antimalarial drug precursor artemisinic acid in engineered yeast. Nature.

[12] Szczebara FM, Chandelier C, Villeret C, Masurel A (2003). Total biosynthesis of hydrocortisone from a simple carbon source in yeast. Nat Biotechnol.

[13] Bennett BD, Kimball EH, Gao M, Osterhout R (2009). Absolute metabolite concentrations and implied enzyme active site occupancy in *Echerichia coli*. Nat Chem Biol.

[14] Sreekumar A, Poisson LM, Rajendiran TM, Khan AP (2009). Metabolomic profiles delineate potential role for sarcosine in prostate cancer progression. Nature.

[15] Frendt SM, Buescher JM, Rudroff F, Picotti P (2010). Tradeoff between enzyme and metabolite efficiency maintains metabolic homeostasis upon perturbations in enzyme capacity. Mol Syst Biol..

[16] Okumoto S, Looger LL, Micheva KD, Reimer RJ, et al Detection of glutamate release from neurons by genetically encoded surface-displayed FRET nanosensors. *Proc Natl Acad Sci USA.* 2005; 102: 8740–8745. 10.1073/pnas.0503274102 PMC1143584 15939876

[17] Mohsin M, Abdin MZ, Nischal L, Kardam H, Ahmad A (2013). Genetically encoded FRET-based nanosensor for in vivo measurement of leucine. Biosens Bioelectron.

[18] Miyawaki A, Llopis J, Heim R, Mc Caffery JM (1997). Fluorescent indicators for Ca^2+^ based on green fluorescent proteins and calmodulin. Nature.

[19] de Lorimier RM, Smith JJ, Dwyer MA, Looger LL (2002). Construction of a fluorescent biosensor family. Protein Sci.

[20] Silva DA, Domınguez-Ramırez L, Rojo-Domınguez A, Sosa-Peinado A (2011). Conformational dynamics of l-lysine, l-arginine, l-ornithine binding protein reveals ligand-dependent plasticity. Proteins..

[21] Hong G, Sylvie L, Okumoto S, Looger LL, Scharff-Poulsen AM (2006). A novel analytical method for in vivo phosphate tracking. FEBS Lett.

[22] Nikaido K, Ames GFL (1992). Purification and characterization of the periplasmic lysine-, arginine-, ornithine-binding protein (LAO) from Salmonella typhimurium. J Biol Chem.

[23] Ha B, Pandit J, Kang C-H, Nikaido K, Gokcen S, Kim S (1993). Three-dimensional structures of the periplasmic lysine/arginine/ornithine—binding protein with and without a ligand. J Biol Chem.

[24] Fehr M, Frommer WB, Lalonde S (2002). Visualization of maltose uptake in living yeast cells by fluorescent nanosensors. Proc Natl Acad Sci USA.

[25] Lager I, Fehr M, Frommer WB, Lalonde S (2003). Development of fluorescent nanosensor for ribose. FEBS Lett.

[26] Kaper T, Looger LL, Takanaga H, Platten M, Steinman L, Frommer WB (2007). Nanosensor detection of an immunoregulatory tryptophan influx/kynurenine efflux cycle. PLoS Biol.

[27] Mohsin M, Ahmad A (2014). Genetically-encoded nanosensor for quantitative monitoring of methionine in bacterial and yeast cells. Biosens Bioelectron.

[28] Kang CH, Shin WC, Yamagata Y, Gokcen S, Ames GF, Kim SH (1991). Crystal structure of the lysine- arginine- ornithine-binding protein (LAO) from *Salmonella typhimurium* at 2.7-A resolution. J Biol Chem.

[29] Lukkarinen M, Nanto-Salonen K, Pulkki K, Aalto M, Simell O (2003). Oral supplementation corrects plasma lysine concentrations in lysinuric protein intolerance. Metabolism..

[30] Burkovski A, Kramer R (2002). Bacterial aminoacid transport: occurrence, functions and significance for biotechnological applications. Appl Microbiol Biotechnol.

[31] Zhang J, Campbell RE, Ting AY, Tsien RY (2002). Creating new fluorescent probe for cell biology. Nat Rev Mol Cell Bio..

[32] Bogner M, Ludewig U (2007). Visualization of arginine flux into plant cells using a specific FRET-sensor. J Fluoresc..

[33] Bradford MM (1976). Rapid and sensitive method for the quantitation of microgram quantities of protein utilizing the principle of protein-dye binding. Anal Biochem.

[34] Laemmli VK (1970). Determination of protein molecular weight in polyacrylamide gels. Nature.

[35] Gruenwald K, Holland JT, Stromberg V, Ahmad A, Watcharakichkorn D, Okumoto S (2012). Visualization of glutamine transporter activities in living cells using genetically encoded glutamine sensors. PLoS One.

[36] Kaper T, Lager I, Looger LL, Chermak D, Frommer WB (2008). Fluorescence resonance energy transfer sensors for quantitative monitoring of pentose and disaccharide accumulation in bacteria. Biotechnol Biofuels.

