# Supplementary material for: Designing, construction and characterization of genetically encoded FRET-based nanosensor for real time monitoring of lysine flux in living cells
Source: J Nanobiotechnology. 2016 Jun 22;14:49. doi: 10.1186/s12951-016-0204-y (PMC4917951; doi:10.1186/s12951-016-0204-y)
Supplement: Supplementary file 1 — 10.1186/s12951-016-0204-y pH stability of the FRET signal of FLIPK. Fluorescent intensity ratio of sensor protein is more stable with PBS buffer whereas in other buffers it is fluctuating. Sensor protein was prepared in Tris-HCl buffer, Phosphate buffer (PB), Tris-Buffered Saline (TBS), Phosphate-BufferedSaline (PBS), HEPES buffer and MOPS buffer at pH 5-8.5 as indicated in the absence of lysine (A) and in the presence of lysine (1 mM) (B). Values are means of three independent replicates. Vertical bars indicate the standard error. [file 12951_2016_204_MOESM1_ESM.docx]

**Additional file 1**. pH stability of the FRET signal of FLIPK

Fluorescent intensity ratio of sensor protein is more stable with PBS buffer whereas in other buffers it is fluctuating. Sensor protein was prepared in Tris-HCl buffer, Phosphate buffer (PB), Tris-Buffered Saline (TBS), Phosphate-BufferedSaline (PBS), HEPES buffer and MOPS buffer at pH 5-8.5 as indicated in the absence of lysine (A) and in the presence of lysine (1 mM) (B). Values are means of three independent replicates. Vertical bars indicate the standard error.
