# Supplementary material for: Designing, construction and characterization of genetically encoded FRET-based nanosensor for real time monitoring of lysine flux in living cells
Source: J Nanobiotechnology. 2016 Jun 22;14:49. doi: 10.1186/s12951-016-0204-y (PMC4917951; doi:10.1186/s12951-016-0204-y)
Supplement: Supplementary file 2 — 10.1186/s12951-016-0204-y Redesign of lysine-binding site for a wide physiological range of detection of lysine and an enhanced response. Predicted structures of different binding site variants in LAOBP. Mutated residues are shown in yellow: Y14A, R77L, F52A, S72A and D161I. [file 12951_2016_204_MOESM2_ESM.docx]

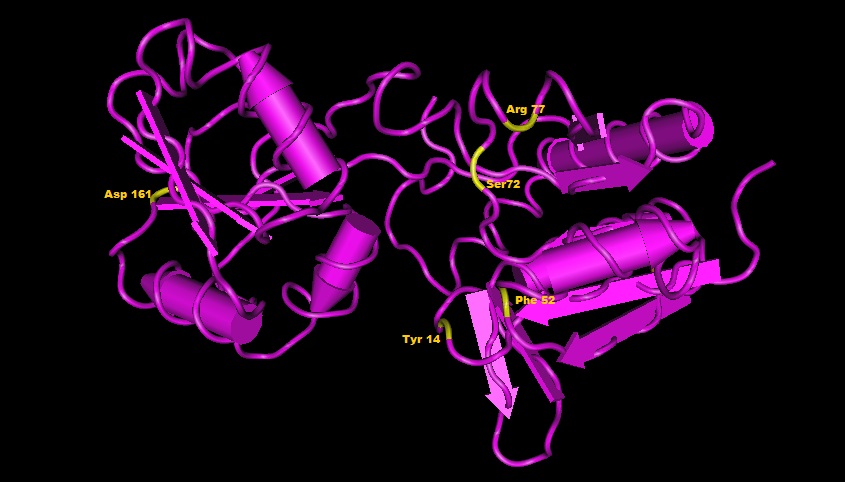


**Additional file 2.**  Redesign of lysine-binding site for a wide physiological range of detection of lysine and an enhanced response. Predicted structures of different binding site variants in LAOBP. Mutated residues are shown in yellow: Y14A, R77L, F52A, S72A and D161I.
