# Supplementary material for: Designing, construction and characterization of genetically encoded FRET-based nanosensor for real time monitoring of lysine flux in living cells
Source: J Nanobiotechnology. 2016 Jun 22;14:49. doi: 10.1186/s12951-016-0204-y (PMC4917951; doi:10.1186/s12951-016-0204-y)
Supplement: Supplementary file 3 — 10.1186/s12951-016-0204-y Confocal imaging of bacterial cells [E. coli BL21 (DE3)] expressing the FLIPK.Scale bar 5 μm. Sensor protein production in the bacterial cells and CFP, YFP and CFP+YFP merged indicating the specific excitation and emission wavelength of the fluorophores. Dual emission intensity ratio was recorded by using LAS-AF software (Leica, Wetzlar, Germany) with 436 nm /20 nm excitation, two emission filters i.e. 480 nm /40 nm for CFP and 535/30 nm for YFP. [file 12951_2016_204_MOESM3_ESM.docx]

**
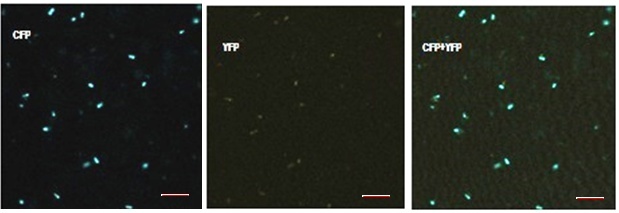
**

**Additional file 3**. Confocal imaging of bacterial cells [*E. coli* BL21 (DE3)] expressing the FLIPK.Scale bar 5 μm. Sensor protein production in the bacterial cells and CFP, YFP and CFP+YFP merged indicating the specific excitation and emission wavelength of the fluorophores. Dual emission intensity ratio was recorded by using LAS-AF software (Leica, Wetzlar, Germany) with 436 nm /20 nm excitation, two emission filters i.e. 480 nm /40 nm for CFP and 535/30 nm for YFP.
