# Supplementary material for: Designing, construction and characterization of genetically encoded FRET-based nanosensor for real time monitoring of lysine flux in living cells
Source: J Nanobiotechnology. 2016 Jun 22;14:49. doi: 10.1186/s12951-016-0204-y (PMC4917951; doi:10.1186/s12951-016-0204-y)
Supplement: Supplementary file 4 — 10.1186/s12951-016-0204-y Confocal imaging of yeast (S. cerevisiae /URA3 strain BY4742) with the time. After addition of 100 mM lysine (indicated by an arrow) the intensity of YFP increases and there is decrease in CFP emission intensity showing that the lysine is transported into the yeast cytosol, where it is recognized by FLIPK. [file 12951_2016_204_MOESM4_ESM.docx]

**Additional file 4.** Confocal imaging of yeast (*S. cerevisiae* /URA3 strain BY4742) with the time. After addition of 100 mM lysine (indicated by an arrow) the intensity of YFP increases and there is decrease in CFP emission intensity showing that the lysine is transported into the yeast cytosol, where it is recognized by FLIPK.
