# Supplementary material for: Designing, construction and characterization of genetically encoded FRET-based nanosensor for real time monitoring of lysine flux in living cells
Source: J Nanobiotechnology. 2016 Jun 22;14:49. doi: 10.1186/s12951-016-0204-y (PMC4917951; doi:10.1186/s12951-016-0204-y)
Supplement: Supplementary file 5 — 10.1186/s12951-016-0204-y Non-normalized and normalized data of visual dynamic of lysine concentration change in the cytosol of yeast. [file 12951_2016_204_MOESM5_ESM.docx]

**
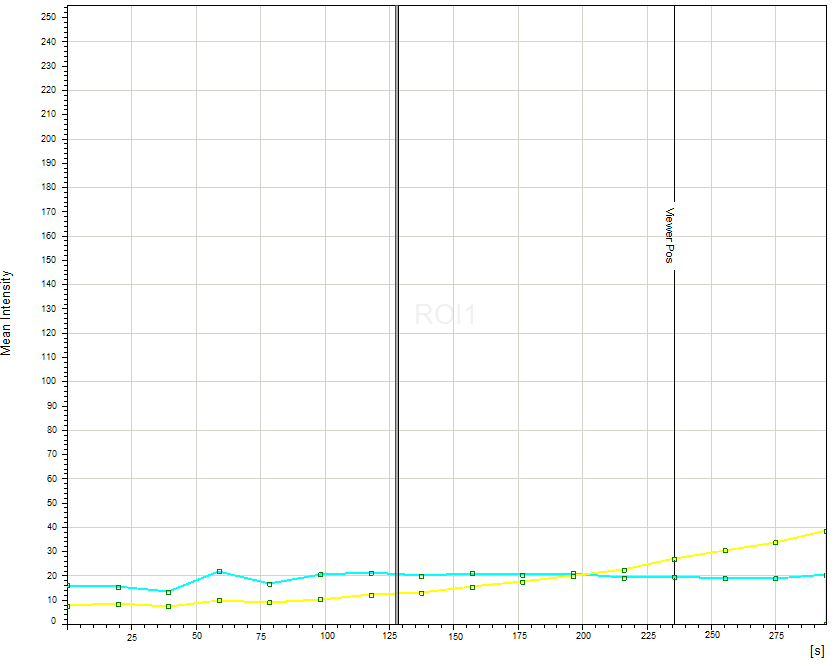
**

Non-normalized data


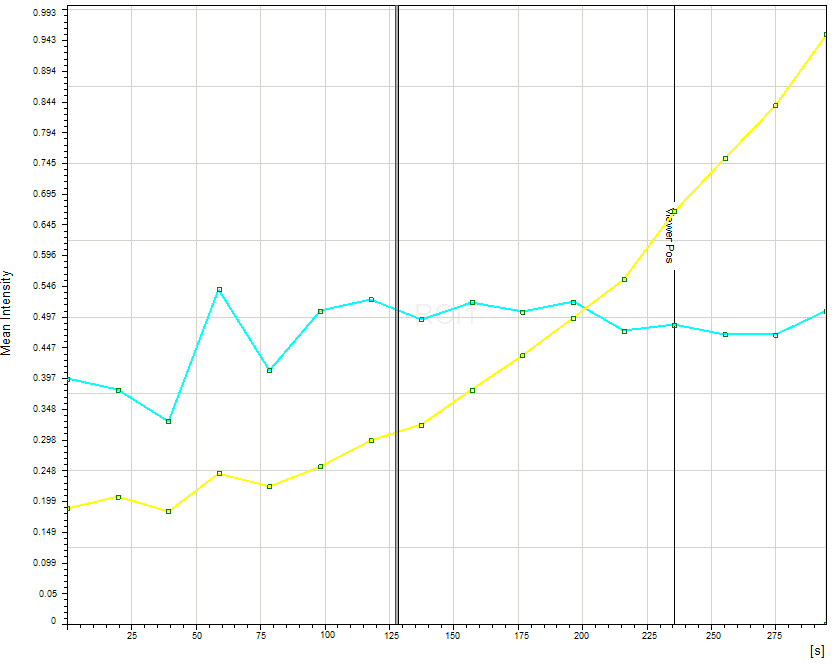


Normalized data

**Additional file 7:**  Non-normalized and normalized data of visual dynamic of lysine concentration change in the cytosol of yeast.
