# Supplementary material for: Designing, construction and characterization of genetically encoded FRET-based nanosensor for real time monitoring of lysine flux in living cells
Source: J Nanobiotechnology. 2016 Jun 22;14:49. doi: 10.1186/s12951-016-0204-y (PMC4917951; doi:10.1186/s12951-016-0204-y)
Supplement: Supplementary file 6 — 10.1186/s12951-016-0204-y A. Nucleotide sequences of construct with His-tag (green)- CFP (cyan) –LAO (grey) –YFP (yellow). B. Amino acid sequence of FLIPK construct. [file 12951_2016_204_MOESM6_ESM.docx]

**A. Nucleotide Sequence**

CATCATCATCATCATCATGGTATGGCTAGCATGACTGGTGGACAGCAAATGGGTCGGGATCTGTACGACGATGACGATAACGCGGATCCAAGTAAAGGAGAAGAACTTTTCACTGGAGTTGTCCCAATTCTTGTTGAATTAGATGGTGATGTTAATGGGCACAAATTTTCTGTCAGTGGAGAGGGTGAAGGTGATGCAACATACGGAAAACTTACCCTTAAATTTATTTGCACTACTGGAAAACTACCTGTTCCATGGCCAACACTTGTCACTACTTTGACTTGGGGTGTTCAATGCTTTTCAAGATACCCAGATCATATGAAACGGCATGACTTTTTCAAGAGTGCCATGCCCGAAGGTTATGTACAGGAAAGAACTATATTTTTCAAAGATGACGGGAACTACAAGACACGTGCTGAAGTCAAGTTTGAAGGTGATACCCTTGTTAATAGAATCGAGTTAAAAGGTATTGATTTTAAAGAAGATGGAAACATTCTTGGTCACAAATTGGAATACAACTATATTTCACACAATGTATACATCACTGCAGACAAACAAAAGAATGGAATCAAAGCTCATTTCAAAATTAGACACAATATTGAAGATGGAAGCGTTCAACTAGCAGACCATTATCAACAAAATACTCCAATTGGCGATGGCCCTGTCCTTTTACCAGACAACCATTACCTGTCCACACAATCTGCCCTTTCGAAAGATCCCAACGAAAAGAGAGACCACATGGTCCTTCTTGAGTTTGTAACAGCTGCTGGGATTACACATGGCATGGATGAACTATACAAAAGCTTGGGGCGCTCCCGCAAACGGTTCGTATTGGAACAGATACCACCTACGCGCCTTTCTCATCAAAAGATGCCAGAGGCGAGTTTATTGGCTTTGATATCGATCTCGGTAATGAAGTGTGTAAGCGTATGCAGGTCAAATGTACCTGGGTCGCCAGCGACTTTGATGCGCTTATTCCCTCACTCAAAGCGAAAAAAATTGATGCCATTATTTCATCGCTCTCTATCACCGATAAACGCCAGCAAGAAATTGCGTCTTCCGACAAACTTTACGCGGCGGATTCACGCCTCATTGCGGCAAAAGGGTCTCCCATTCAGCCGACGCTGGAATCGCTCAAAGGCAAGCATGTCGGCGTGCTGCAAGGGTCCACGCAAGAGGCTTACGCCAATGATAACTGGCGCACAAAAGGTGTGGATGTGGTGGCTTATGCCAACCAGGATCTTATCTATTCCGATTTCACCGCCGGTCGTCTGGATGCCGCATTGCAGGATGAAGTCGCCGCCAGCGAAGGTTTCCTCAAGCAGCCAGCGGGCAAAGAGTATGCATTTGCCGGTCCTTCCGTCAAGGATAAAAAATATTTTGGCGACGGGACGGGGGTTGGGCTGCGCAAAGACGATACCGAGCTACAAGCCGCGTTTGATAAAGCGCTCACCGAACTGCGCCAGGACGGAACTTACGACAAAATGGCCAAAAAGTACTTCGATTTTAATGTTTACGGCGATTGACCGGGACTTGTCGAGGAGAAGAACTTTTCACTGGAGTTGTCCCAATTCTTGTTCAATTAGATGGTCATGTTCATGGGCACAAATTTTCTGTCAGTGGAGAGGGTCAAGGTCATGCAACATACGGAAAACTTACCCTTCAATTTATTTGCACTACTGGAAAACTACCTGTTCCATGGCCAACACTTGTCACTACTTTCGGTTATGGTCTAAAATGCTTTGCTGGATACCCAGATCATATGAAACGGCATCACTTTTTCAAGAGTGCCATGCCCGAAGGTTATGTACAGGAAAGAACTATATTTTTCAAAGATCACGGGAACTACAAGACACGTGCTCAAGTCAAGTTTCAAGGTCATACCCTTGTTCATCGAATCGAGTTAAAAGGTATTACTTTTACAGAAGATGGAAACATTCTTGGACACAAATTGGAATACAACTATCACTCACACAATGTATACATCATGGCAGACAAACAAAAGAATGGAATCAAAGTTCACTTCAAAATTCGACACAACATTCAAGATGGAAGCGTTCAACTAGCAGACCATTATCAACAAAATACTCCAATTGGCGATGGCCCTGTCCTTTTACCAGACAACCATTACCTGTCCTATCAATCTGCCCTTTCGAAAGATCCCAACGAAAAGAGAGACCACATGGTCCTTCTTGcGTTTGTAACAGCTGCTGGGATTACACATGGCATGGATGCACTATACAAAGAGCTCGAATC

**B. Amino acid sequence**

H H H H H H G M A S M T G G Q Q M G R D L Y D D D D N A D P S K G E E L F T G V V P I L V E L D G D V N G H K F S V S G E G E G D A T Y G K L T L K F I C T T G K L P V P W P T L V T T L T W G V Q C F S R Y P D H M K R H D F F K S A M P E G Y V Q E R T I F F K D D G N Y K T R A E V K F E G D T L V N R I E L K G I D F K E D G N I L G H K L E Y N Y I S H N V Y I T A D K Q K N G I K A H F K I R H N I E D G S V Q L A D H Y Q Q N T P I G D G P V L L P D N H Y L S T Q S A L S K D P N E K R D H M V L L E F V T A A G I T H G M D E L Y K S L G R S R K R F V L E Q I P P T R L S H Q K M P E A S L L A L I S I S V M K C V S V C R S N V P G S P A T L M R L F P H S K R K K L M P L F H R S L S P I N A S K K L R L P T N F T R R I H A S L R Q K G L P F S R R W N R S K A S M S A C C K G P R K R L T P M I T G A Q K V W M W W L M P T R I L S I P I S P P V V W M P H C R M K S P P A K V S S S S Q R A K S M H L P V L P S R I K N I L A T G R G L G C A K T I P S Y K P R L I K R S P N C A R T E L T T K W P K S T S I L M F T A I D R D L S R R R T F H W S C P N S C S I R W S C S W A Q I F C Q W R G S R S C N I R K T Y P S I Y L H Y W K T T C S M A N T C H Y F R L W S K M L C W I P R S Y E T A S L F Q E C H A R R L C T G K N Y I F Q R S R E L Q D T C S S Q V S R S Y P C S S N R V K R Y Y F Y R R W K H S W T Q I G I Q L S L T Q C I H H G R Q T K E W N Q S S L Q N S T Q H S R W K R S T S R P L S T K Y S N W R W P C P F T R Q P L P V L S I C P F E R S Q R K E R P H G P S C V C N S C W D Y T W H G C T I Q R A R I

**Additional file 5:**

A. Nucleotide sequences of construct with His-tag (green)- CFP (cyan) –LAO (grey) –YFP (yellow).

B. Amino acid sequence of FLIPK construct
