# Supplementary material for: Designing, construction and characterization of genetically encoded FRET-based nanosensor for real time monitoring of lysine flux in living cells
Source: J Nanobiotechnology. 2016 Jun 22;14:49. doi: 10.1186/s12951-016-0204-y (PMC4917951; doi:10.1186/s12951-016-0204-y)
Supplement: Supplementary file 7 — 10.1186/s12951-016-0204-y SDS-PAGE of expressed FLIPK in E. coli BL21 (DE3). M= protein marker; L1= uninduced protein sample; L2= induced protein sample. [file 12951_2016_204_MOESM7_ESM.docx]

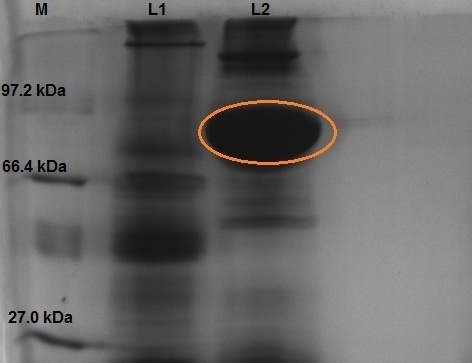


**Additional file 6:** SDS-PAGE of expressed FLIPK in *E. coli* BL21 (DE3). M= protein marker; L1= uninduced protein sample; L2= induced protein sample.
